# Advanced NSCLC with leptomeningeal metastasis: Diagnostic and therapeutic challenges: A case report

**DOI:** 10.1016/j.radcr.2025.08.004

**Published:** 2025-08-26

**Authors:** Gana Odeh, Dana Foqha, Mohammed Abu Kamesh, Jihad Hamaida

**Affiliations:** a Medical Intern, An-Najah National University Hospital, Nablus, Palestine; b Radiology Resident, Department of Radiology, An-Najah National University Hospital, Nablus, Palestine; c Radiologist Consultant, Head of Radiology Department, An-Najah National University Hospital, Nablus, Palestine

**Keywords:** Lung cancer, NSCLC, Leptomeningeal metastasis, Brain metastasis, Crizotinib

## Abstract

Lung cancer, particularly non-small lung cancer (NSCLC), is one of the most common malignant tumors worldwide and is associated with high mortality in advanced stages. Lung adenocarcinoma is the most frequent NSCLC subtype. Among these common metastatic sites, brain metastases and leptomeningeal metastases have a particularly debilitating impact on patients’ survival and quality of life. We describe the clinical course of a 43-year-old man with stage IV lung adenocarcinoma who developed crizotinib-induced pancreatitis and leptomeningeal metastases, both are rare. Imaging of the brain was done due to the development of neurological symptoms and demonstrated metastatic leptomeningeal enhancement. The patient’s condition continued to worsen and resulted in sudden cardiac arrest. This case demonstrates a rare complication of NSCLC—leptomeningeal metastasis—and the importance of a multidisciplinary team approach in managing advanced NSCLC.

## Introduction

Lung cancer remains the most common malignancy leading to death, with NSCLC making up the majority of cases [1,2]. Lung adenocarcinoma is the most common subtype of NSCLC, accounting for 85% of lung cancer cases [3]. Advanced cases are associated with poor prognosis; even with chemotherapy, only 10%-20% of NSCLC patients survive beyond 2 years. Up to 20% of NSCLC patients have brain metastases at the time of diagnosis, and 25%-40% of patients develop brain metastases along the course of the disease [4].

Another serious complication of NSCLC metastasis is leptomeningeal carcinomatosis (LMC), where cancer cells reach the pia mater, arachnoid, subarachnoid space, and cerebrospinal fluid (CSF) spaces [5]. The reported incidence of LMC in patients with advanced NSCLC ranges between 3% and 5%. Clinical manifestations of LMC are highly variable, and its early diagnosis is challenging [6].

Here, we report a case of a 43-year-old patient with LMC, including the presentation, diagnostic methods, and management, showcasing the importance of a multidisciplinary approach in handling such cases.

## Case summary

**Fig. 1:**
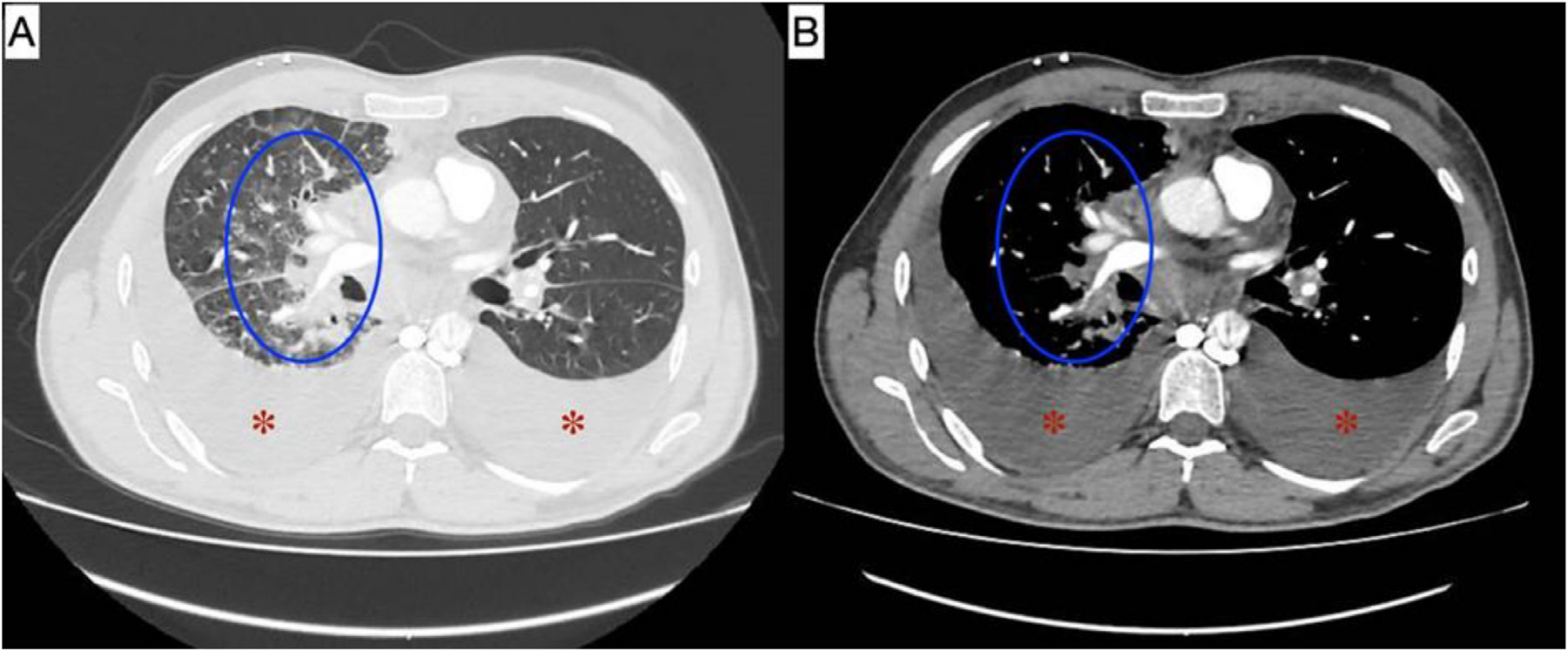
(A) Lung window CT; (B) Mediastinal window CT. Blue circles represent areas of perihilar consolidation accompanied by surrounding ground-glass opacities and architectural distortion, which are characteristic radiological features of lung adenocarcinoma. Burgundy asterisks denote the presence of bilateral pleural effusion in both windows.

**Fig. 2:**
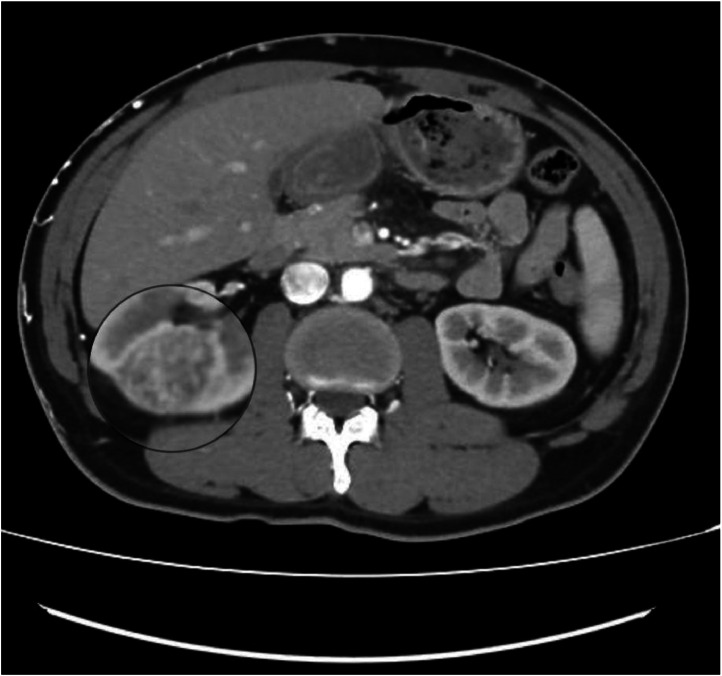
Coned view of a venous-phase CT at the level of the mid-portion of the right kidney, demonstrating a well-defined, heterogeneously enhancing renal cortical lesion. These imaging features are suggestive of either renal cell carcinoma or metastatic deposits. (However, no further investigations were conducted to confirm the diagnosis).

We present a case of a 43-year-old male patient with stage IV lung adenocarcinoma, diagnosed within the past year (Fig. 1). Alongside, he was found to have a right renal mass suspected to be either renal cell carcinoma or a metastatic lesion (Fig. 2). He had been managed with 6 cycles of carboplatin-paclitaxel chemotherapy and was on crizotinib for the past 3 months. His medical history included: tachyarrhythmia, managed with bisoprolol for 7 years; a history of deep vein thrombosis (DVT) in the left subclavian vein, for which he was on apixaban; and recurrent malignant pleural effusion requiring frequent drainage. His social history includes a 10-pack-year smoking history.

**Fig. 3:**
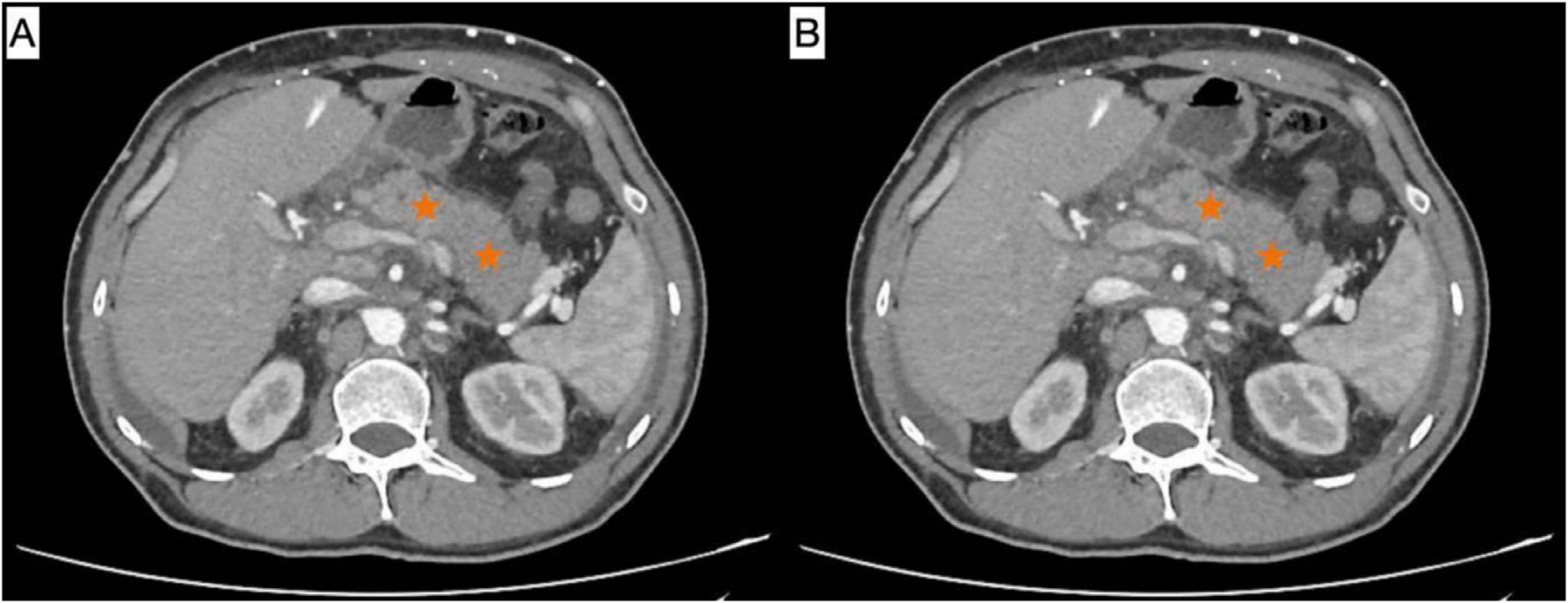
Contrast-enhanced axial CT images in soft tissue window, (A) arterial phase; (B) venous phase. The pancreas (orange stars) appears enlarged and edematous, with surrounding fat stranding and regional lymphadenopathy—findings consistent with acute pancreatitis.

The patient was admitted with abdominal pain lasting 10 days and vomiting for 1 day. Labs showed an amylase of 285 U/L and a lipase of 510 U/L. Computed tomography (CT) revealed a bulky head of the pancreas and uncinate process surrounded by diffuse fat stranding and regional lymph nodes, suggestive of pancreatitis (Fig. 3). Pancreatitis was suspected to be drug-induced (crizotinib-related) and led to discontinuation of the medication. He was managed effectively for his complaint and discharged; however, 3 days later, he was seen in the emergency department (ED) with mild epigastric tenderness which improved after supportive management.

**Fig. 4:**
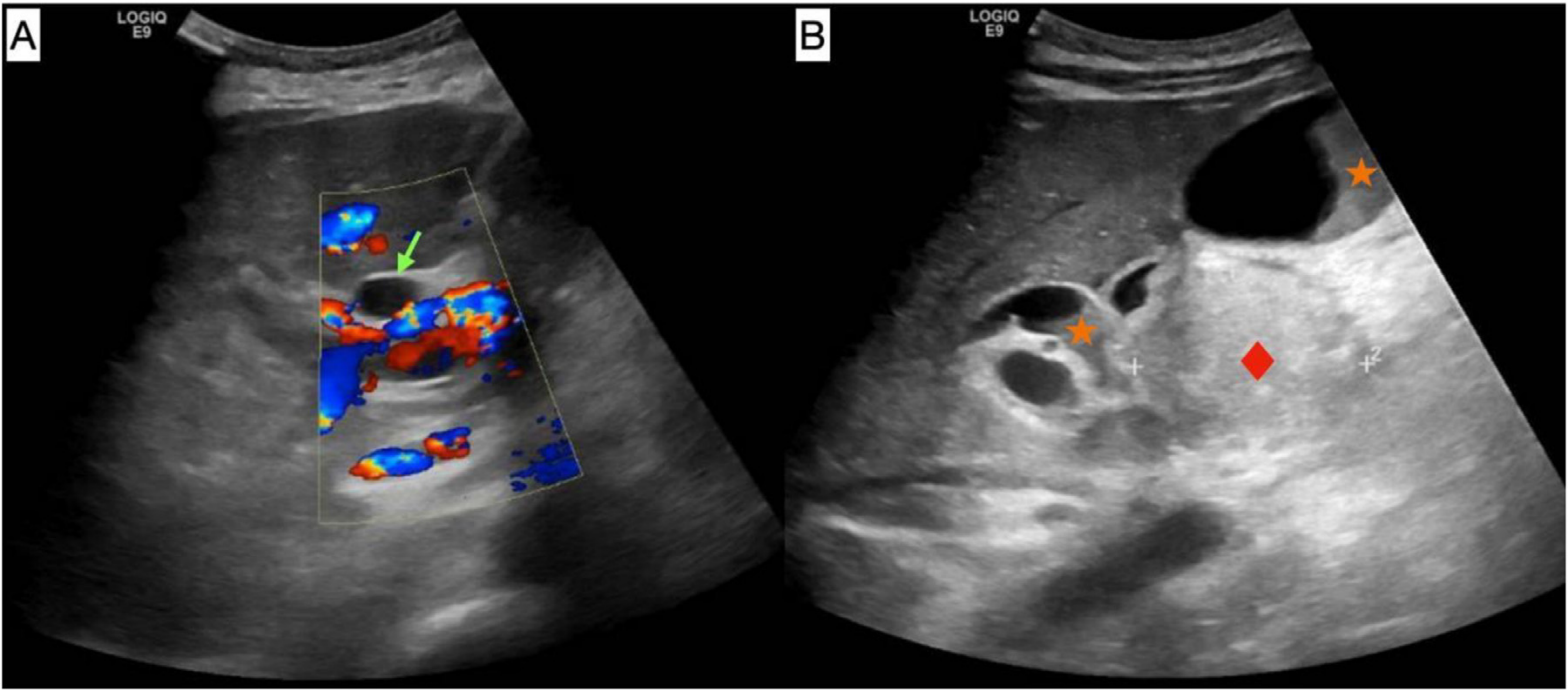
Selected transabdominal ultrasound images at the level of (A) Porta hepatis; (B) Pancreatic head. The common bile duct (CBD) (light green arrow) appears dilated, measuring approximately 0.9 cm, likely due to extrinsic compression by the inflamed and edematous pancreatic head (red diamond). This results in minimal intrahepatic biliary dilatation. Thick echogenic sludge is visualized within the gallbladder and distal CBD (orange stars).

Following this event, the patient was readmitted fifteen days later due to obstructive jaundice caused by acute cholangitis, most likely due to malignant obstruction. The patient had jaundice with elevated total and direct bilirubin levels: total bilirubin 4.05 mg/dL and direct bilirubin 2.93 mg/dL. Abdominal ultrasound showed dilated common bile duct measuring 0.9 cm and faint intrahepatic biliary tree dilatation (Fig. 4). Endoscopic Retrograde Cholangiopancreatography (ERCP) was performed. The ampulla of Vater appeared grossly normal. Cholangiogram showed a long distal common bile duct stricture suggestive of malignant stricture, with gross proximal biliary dilatation. Dilatation was performed, and a self-expanding metallic stent was inserted.

On the same day of admission, the patient was also found to have malignant pleural effusion, and suspected transient ischemic attack (TIA) in the left middle cerebral artery (MCA), with the transient ischemic attack (TIA) resolving within minutes. Additionally, moderate intraperitoneal free fluid was noted. The patient was assessed for definitive management of his recurrent pleural effusion, for which a decision was made by the surgical team to insert chest tubes on both sides.

**Fig. 5:**
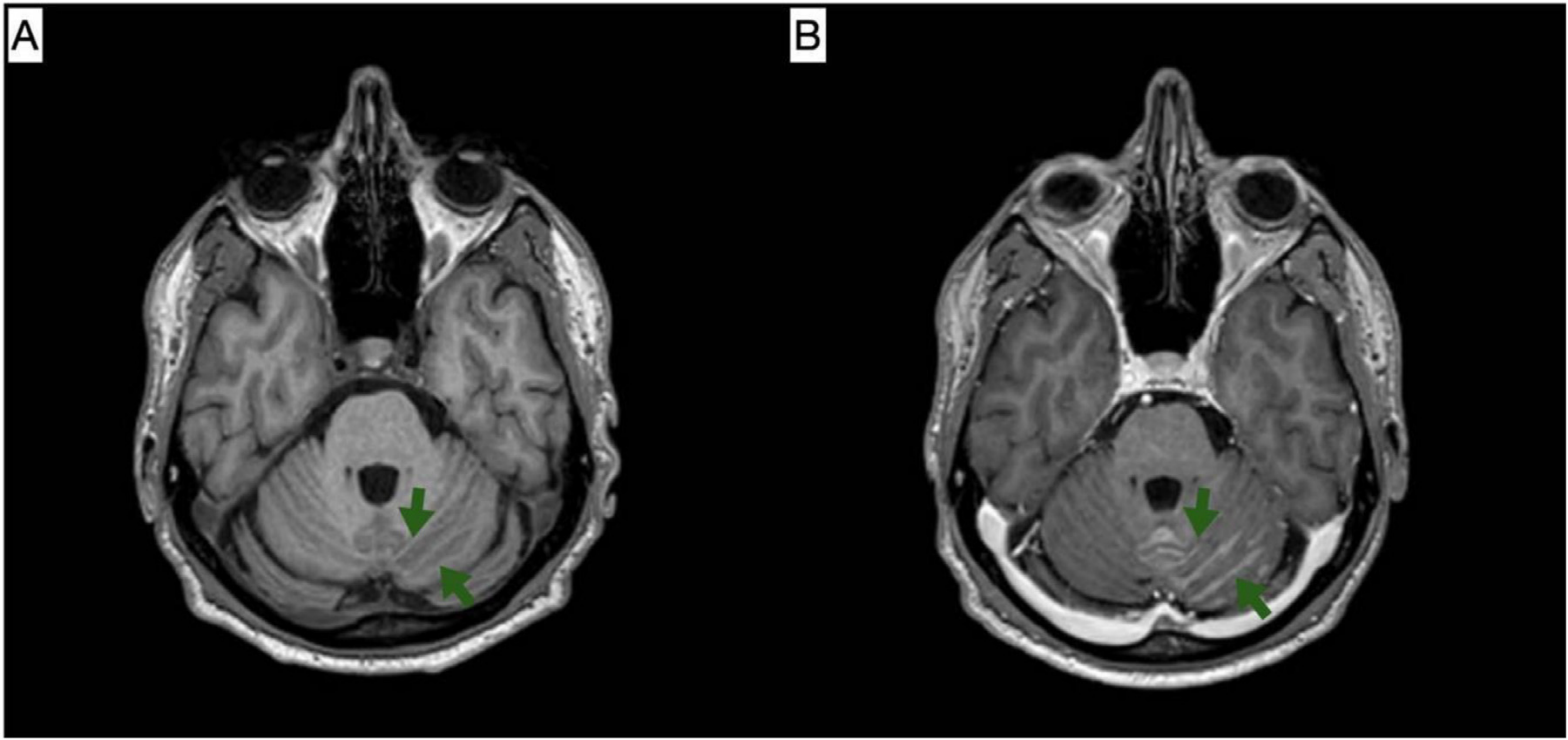
(A) T1-weighted image; (B) T1-weighted image post-contrast. Selected MRI images at the level of the superior cerebellum demonstrate leptomeningeal enhancement of the left cerebellum (indicated by green arrows), which is suggestive of metastatic deposits.

**Fig. 6:**
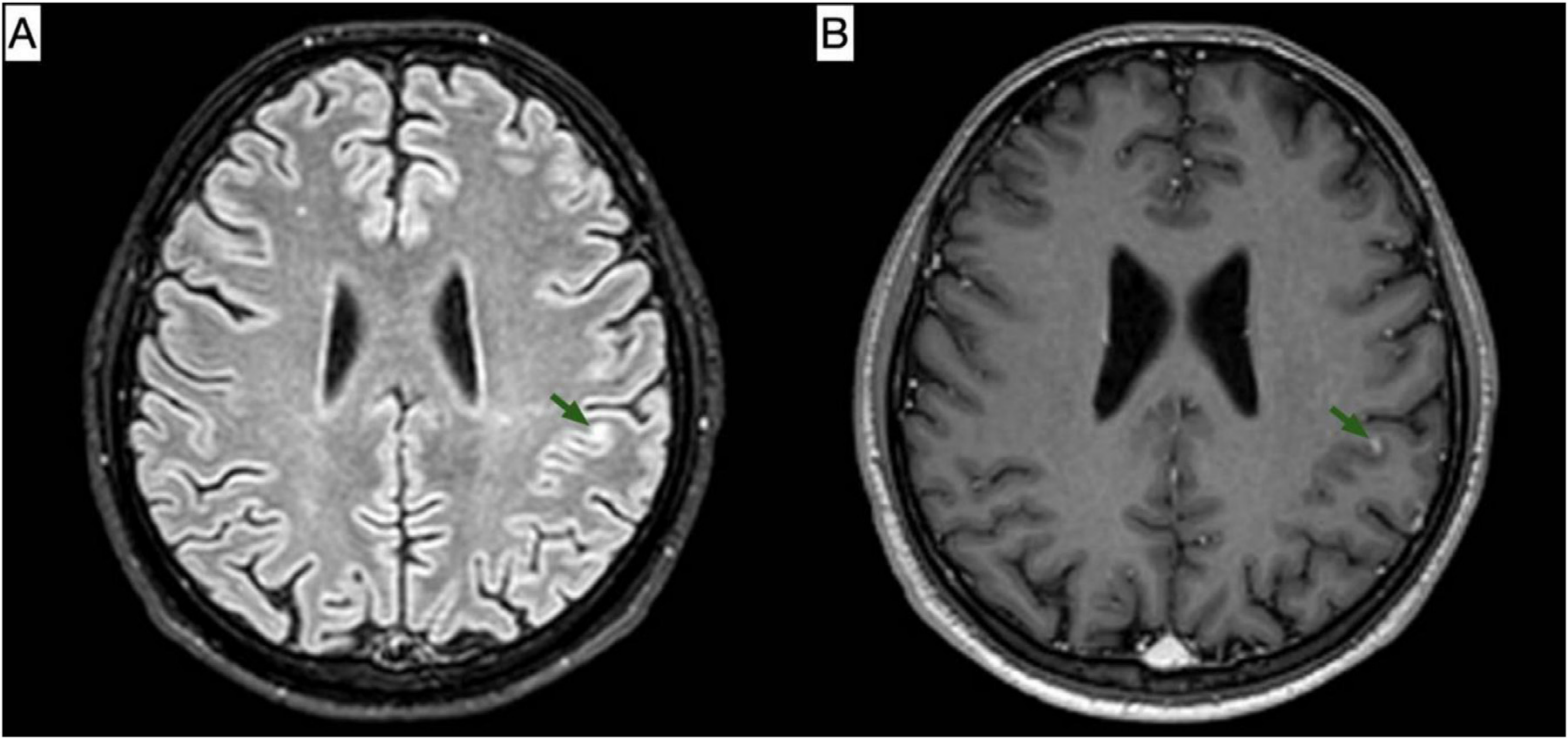
(A) FLAIR sequence; (B) T1-weighted image post-contrast. Selected axial MRI images demonstrate an enhancing focus at the left parietal corticomedullary junction, which appears hyperintense on the FLAIR sequence (indicated by green arrows), suggestive of a metastatic focus.

**Fig. 7:**
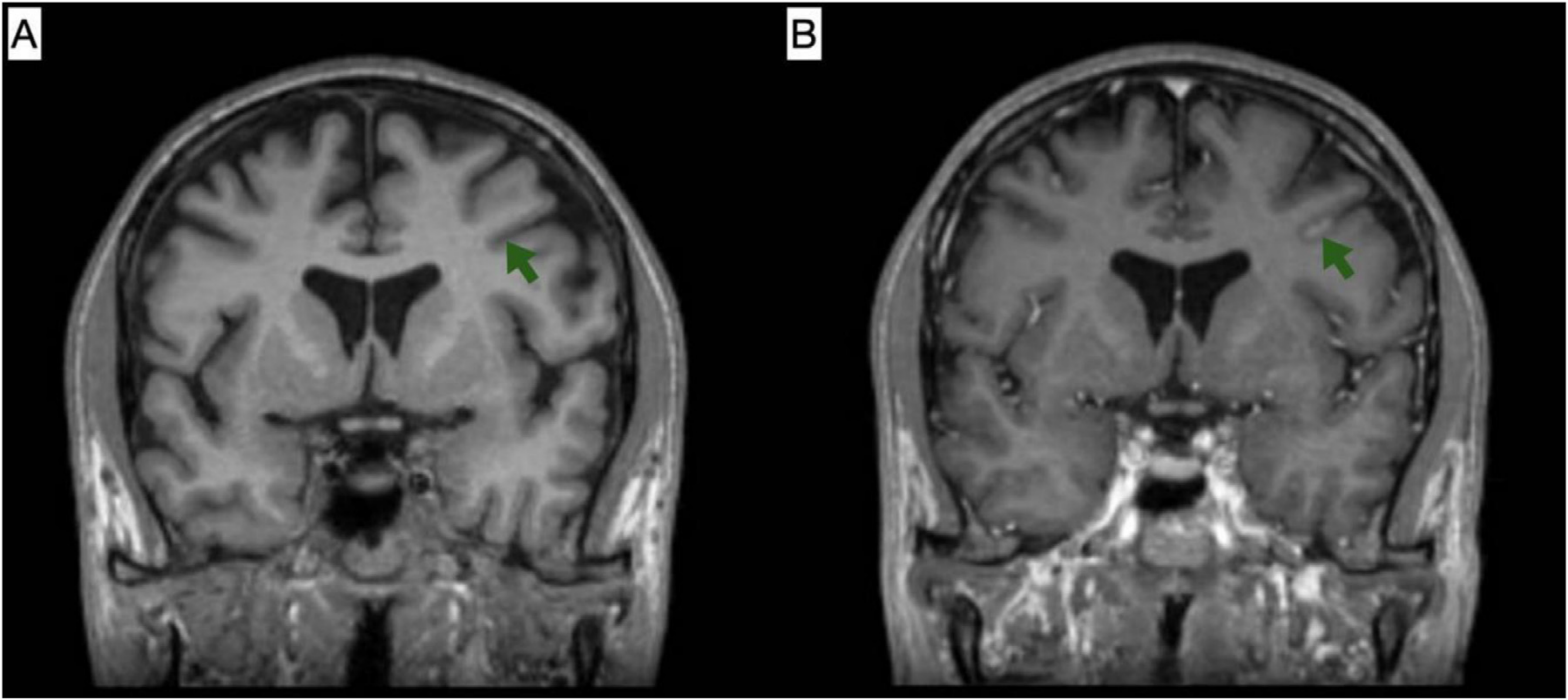
(A) T1-weighted image; (B) T1-weighted image post-contrast. Selected coronal MRI images reveal supratentorial leptomeningeal enhancement of the left cerebrum (indicated by green arrows), suggestive of metastatic deposits.

On the following day, the patient experienced multiple episodes of right-sided paresthesia with slurred speech and involuntary perioral twitching. Upon examination, the patient was conscious, oriented, and alert. The neurological exam showed no motor or sensory weakness, with normal deep tendon reflexes, a negative Babinski sign, and a negative Hoffman sign. The cranial nerve examination was normal except for a congenital right pupil anomaly. The history and symptoms were suggestive of focal seizures. Brain Magnetic resonance imaging (MRI) with contrast and CT scans without contrast were ordered, and the neurology team was consulted. The brain CT showed no abnormalities. The MRI revealed a new appearance of a few supra and infratentorial hyperintense T2/Fluid-attenuated inversion recovery (FLAIR) foci that appeared hypointense on TIWI. Multiple nonspecific hyperintense T2/FLAIR foci were seen scattered in both cerebral hemispheres. The appearance is suggestive of metastatic lesion. It also showed leptomeningeal enhancement on the left side of the cerebellum and, to a lesser extent, in the supratentorial region. These findings suggested leptomeningeal metastasis (Fig. 5, Fig. 6, Fig. 7).

The following day, a Doppler ultrasound of the carotid arteries was performed. The Doppler study was unremarkable. The patient was started on dexamethasone, levetiracetam, and clonazepam.

Neurologically, the patient experienced fewer episodes of right-sided paresthesia after starting levetiracetam and reported no new neurological complaints. The neurological exam the next day was normal, with no motor or sensory deficits, abnormal gait, or other cerebellar signs. An Electroencephalogram (EEG) was performed and showed no abnormalities. Given that brain imaging showed new supra- and infratentorial metastatic lesions with leptomeningeal enhancement on the left side of the cerebellum and, to a lesser extent, in the supratentorial region, the patient remained on follow-up with continued medication.

**Fig. 8:**
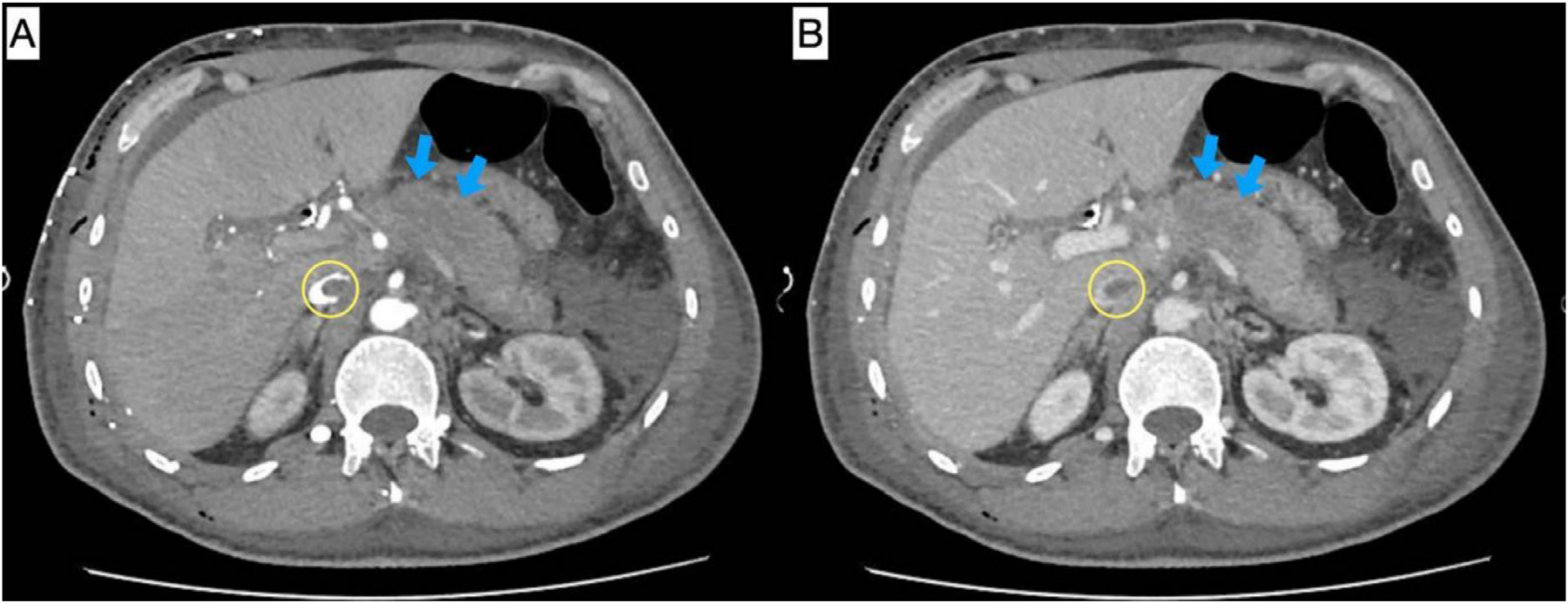
Contrast-enhanced axial CT images in soft tissue window, (A) arterial phase; (B) venous phase. The pancreas (blue arrows), which is heterogeneously enlarged, demonstrates central non-enhancing hypodense areas, consistent with necrosis. Associated findings include peripancreatic fat stranding, upper abdominal free fluid, and regional lymphadenopathy—features suggestive of necrotizing pancreatitis. Yellow circles highlight a filling defect within the inferior vena cava (IVC), suggestive of IVC thrombosis.

A few days after ERCP, the patient complained of severe abdominal pain. He had rising amylase and lipase: 823 U/L and 1076 U/L, respectively. CT scan was done and showed swollen and enlarged pancreas, surrounded by diffuse fat stranding and regional lymph nodes, the largest measures about 1.5 cm in short axis; as well as mild diffuse reactionary wall thickening seen in the pyloric, duodenum and proximal jejunal loops. Features were suggestive of acute pancreatitis. There was a newly developed hypoenhancing areas seen in the head and body on the pancreas suggesting pancreatic necrosis (Fig. 8). The patient’s situation deteriorated with severe pain and respiratory distress and was admitted to the surgical intensive care unit as a case of necrotizing pancreatitis. His situation was complicated by acute kidney injury and hypoxic respiratory failure. After 4 days of management, he was transferred back to the ward. His pancreatitis resolved, but 3 days later, his bilirubin was trending up and he was found to have a stent obstruction, for which he was planned for ERCP. Due to his bilateral pleural effusion, pleurodesis was planned. However, neither procedure was performed, as 4 days later, he developed a new onset left hand weakness (power 0/5) with facial weakness, mouth deviation, and an abnormal gag reflex, raising suspicion of new brain metastases. Because of this, ERCP and pleurodesis were canceled, and the patient was kept on medical management.

The patient’s condition was deteriorating, with increasing oxygen requirements and an increasing International Normalized Ratio (INR). He also had recurrent episodes of coffee-ground emesis, and his chest tubes produced reddish fluids. The patient had a poor prognosis; he and his family were informed by the attending physician, and they agreed to keep the patient on palliative measures. A few days later, the patient had a sudden cardiac arrest. Cardiopulmonary resuscitation was attempted, but the patient did not survive (Table 1, Table 2).

**Table 1:** Timeline of historical and current information.

Timepoint	Event
Past medical history	Tachyarrhythmia (on bisoprolol), DVT of left subclavian vein (on apixaban), recurrent malignant pleural effusions
∼12 months ago	Diagnosis of stage IV lung adenocarcinoma (perihilar consolidation with bilateral pleural effusion)
Same period	Right renal mass discovered (likely RCC or metastasis)
∼3 months ago	Started crizotinib (ALK inhibitor)
Social history	10-pack-year smoking history
Recent admission	Admitted with drug-induced pancreatitis, suspected crizotinib-related
3 days later	Mild epigastric tenderness, resolved with supportive care
15 days later	Readmitted with obstructive jaundice, recurrent pleural effusion, and suspected left MCA TIA (resolved), also noted moderate intraperitoneal free fluid
Same admission	Chest tubes inserted bilaterally for pleural effusion
On the following day	Neurological symptoms (right-sided paresthesia, perioral twitching, slurred speech); brain imaging showed leptomeningeal metastases, supra/infratentorial lesions
Imaging results	CT: normal; MRI: leptomeningeal enhancement (cerebellum and cerebrum), T2/FLAIR foci suggestive of metastasis
Post-imaging	Started on dexamethasone, levetiracetam, clonazepam
EEG	Normal
Few days later	Deterioration: necrotizing pancreatitis, AKI, hypoxic respiratory failure – admitted to SICU
After 4 days	Returned to ward, pancreatitis resolved
3 days later	Increasing bilirubin → stent obstruction identified, planned ERCP
Also planned	Pleurodesis for recurrent effusion
Few days later	Developed new neurological deficits (left hand weakness, facial involvement, abnormal gag) suggesting new brain metastases
Final days	Worsening respiratory status, rising INR, coffee-ground emesis, hemorrhagic chest tube drainage
Final outcome	Transitioned to palliative care, cardiac arrest, unsuccessful resuscitation

**Table 2:** Intervention adherence and tolerability.

Intervention	Adherence	Tolerability
Bisoprolol	Long-term use for tachyarrhythmia	No noted issues
Apixaban	Ongoing for DVT	Risk in context of bleeding and high INR later
Crizotinib	On for 3 months before adverse events	Suspected cause of pancreatitis (major toxicity)
ERCP	Metallic stent was inserted	Necrotizing pancreatitis, a few days later, mostly post-ERCP
Levetiracetam + Clonazepam	Initiated post-seizure-like activity	Controlled further seizures effectively; good tolerability reported
Chest tubes	Inserted bilaterally	Functioned but later led to hemorrhagic output; possible bleeding complication
ERCP and Pleurodesis	Planned but not performed due to worsening neurological condition	-
Dexamethasone	Given for brain edema/metastasis	Presumed effective in symptom control
Palliative care	Patient and family agreed after counseling	Implemented in final phase

## Discussion

This case demonstrates the debilitating nature of non-small cell lung cancer (NSCLC) and the challenging complications that may arise during the course of the disease. From chemotherapy-induced pancreatitis to central nervous system (CNS) metastases, this case shows the multi-organ effects alongside appropriate management of events.

NSCLC is commonly known for spreading to the CNS [7]. Among patients with advanced NSCLC, CNS metastases were documented in 40%-50% [7,8]. These metastases may be located either in the brain parenchyma or in a leptomeningeal distribution [8]. LMC, which was evident in the presented case, is uncommon and reported in only 3%-5% of NSCLC patients [9,10].

LMC can manifest in a variety of non-specific neurological symptoms and is associated with poor outcomes and fatal complications [10,11]. Without treatment, LMC leads to death within 4 to 6 weeks. Even when treated, the prognosis remains poor, with an estimated overall survival of only 2-4 months [12].

The gold standard diagnostic method for LMC is CSF cytology [11]. However, MRI is routinely ordered and can be used alone in the diagnosis process due to its sensitivity [13]. A study published in the *American Journal of Neuroradiology* estimated the sensitivity of MRI at 65% when combining all sequences—unenhanced FLAIR, contrast-enhanced FLAIR, and contrast-enhanced T1-weighted imaging. Contrast-enhanced T1-weighted MRI is the most sensitive imaging sequence for detecting leptomeningeal disease. In contrast, CT scans are less effective in identifying these metastatic lesions [14]. Since neither cytology nor MRI alone is sufficiently sensitive for diagnosing LMC, the diagnostic approach should include serial CSF analysis or clinical suspicion supported by MRI findings [13,15].

This aligns with our case, where a patient with stage IV lung cancer presented with focal seizures. Appropriately, neurological imaging was ordered, including a contrast-enhanced MRI, which showed supratentorial and infratentorial metastatic lesions with leptomeningeal enhancement, suggestive of leptomeningeal metastasis.

For managing the patient’s neurological symptoms, levetiracetam was used, leading to symptom improvement. This medication is effective in reducing seizure frequency with fewer side effects, making it preferred for brain tumor-related epilepsy [16]. Additionally, since cancer patients are often on multiple medications, levetiracetam offers the advantage of minimal drug-drug interactions, as it does not induce the cytochrome P450 system [17].

Before the neurological manifestations, the patient experienced pancreatitis, which was mostly attributed to crizotinib and led to the discontinuation of this medication. This medication, once discovered, revolutionized the management of NSCLC with anaplastic lymphoma kinase (ALK) chromosomal rearrangements [18]. Despite the proven efficacy of this drug, it is associated with a broad range of organ toxicity that is important to monitor [19]. However, the pancreas is not usually mentioned, making the patient’s presentation in this case an unusual association. Additionally, even after following this treatment plan, the patient developed CNS metastases. This was probably due to the poor ability of this drug to accumulate in the CNS [20]. This is consistent with what is mentioned in the literature—metastasis was evidenced in many patients during the first year of using crizotinib [20].

This case, with both parenchymal and leptomeningeal metastases, highlights key considerations in the pathophysiology and diagnostic challenges of NSCLC with CNS involvement. The strength of this case lies in its emphasis on the importance of a multidisciplinary approach. Additionally, the imaging and diagnostic steps were all done correctly and purposefully, as indicated for management in similar cases. Furthermore, ethical decisions such as initiating palliative care were clearly communicated with the family based on the patient’s deterioration. On the other hand, the case was limited by the reduced ability to continue therapeutic interventions due to the rapid deterioration of the patient.

## Conclusion

The case presents the challenges of treating advanced NSCLC, especially when rare complications like leptomeningeal metastasis and crizotinib-induced pancreatitis are involved. It emphasizes the importance of detecting neurological symptoms early and using contrast-enhanced MRI for diagnosis. While crizotinib is effective, it has limited ability to penetrate the CNS. Drug toxicities are expected, but pancreatitis is a rare side effect, making this case particularly unique. Overall, the case reinforces the need for a multidisciplinary approach to ensure timely diagnosis, effective management, and better care for patients with advanced lung cancer.

## Patient consent

Written informed consent was obtained from the patient next of kin (brother) for publication of this case report and any accompanying images.
